# Pupillometry as a measure of cognitive load in mental rotation tasks with abstract and embodied figures

**DOI:** 10.1007/s00426-021-01568-5

**Published:** 2021-08-12

**Authors:** Robert Bauer, Leonardo Jost, Bianca Günther, Petra Jansen

**Affiliations:** Faculty of Human Sciences, University of Regensburg, Universitätsstraße 31, 93053 Regensburg, Germany

## Abstract

We investigated sex differences in behavioral performance and cognitive load in chronometric mental rotation tasks with abstract and embodied figures. Eighty participants (44 females and 36 males) completed 126 items, which included cube figures, body postures, and human figures, which were all comparable in shape and color. Reaction time, accuracy, and cognitive load, measured by changes in pupil dilation, were analyzed. As a function of angular disparity, participants showed shorter reaction times and higher accuracy rates for embodied stimuli than cube figures. Changes in pupil dilation showed a similar pattern, indicating that mental rotation of embodied figures caused less cognitive load to solve the task. No sex differences appeared in any of the measurements.

## Introduction

In everyday activities, spatial abilities play an important role, for instance, in navigation, natural sciences, and engineering. They are related to various abilities such as mathematical mathematical ability (Xie et al., 2019) or problem solving (Geary et al., 2000), among others. Uttal et. al. (2013) differentiate spatial abilities according to two dimensions: extrinsic versus intrinsic and static versus dynamic. Mental rotation is an intrinsic dynamic ability that describes the ability to mentally rotate 2D or 3D objects fast and accurately (Linn & Petersen, 1985; Shepard & Metzler, 1971). Two mental rotation tasks are often used: an object-based or egocentric mental rotation (Zacks et al., 2000). An existing assumption is that pictures of abstract or non-human objects, like cube figures, are processed with an object-based mental transformation. In contrast, human body or body parts pictures are assumed to be embodied, and therefore, to be processed with an egocentric perspective-based mental transformation (Zacks & Tversky, 2005).

Mental rotation experiments use human body figures to evoke motor resonance processes, which would lead to better task performance by utilizing a sensorimotor simulation mechanism (e.g., Buccino et al., 2004; Calvo-Merino et al., 2005; Liuzza et al., 2012; Voyer & Jansen, 2016). This mechanism is also supported by behavioral and neuroimaging data indicating similar motor representations between observing, performing and mentally imagining an action (for reviews, see Decety, 2002; Dijkstra & Post, 2015). The findings of Amorim et. al. (2006) indicate that the stimulus type is essential, due to increased familiarity, e.g. by adding a human head to cube figures. They further conclude that familiar postures would be easier to emulate than unfamiliar or atypical ones, eliciting embodied spatial transformations that facilitate task performance. The use of pictures of human bodies or body parts (e.g. pictures of hands) as stimuli in mental rotations tasks results from the embodied cognition approach (Lakoff & Johnson, 1999).

## Object-based versus egocentric transformations

In an object-based transformation task, the participants have to decide whether or not two objects, which are rotated to each other, are mirror reversed to each other (then they are called “different”) or not (then they are called “same”). In an egocentric transformation, e.g. using human figures with raised arms (or hand pictures), participants have to determine if it is the left or right arm (hand). Both transformation types are connected to different cognitive processes. In the object-based transformation, the observer’s position stays fix while mentally moving the object relative to its surroundings. In the egocentric one, a participant changes his perspective and mentally rotates his own body relative to the object (Voyer et al., 2017). Unfortunately, egocentric and object-based mental rotation tasks confound the stimulus type (embodied versus non-embodied) and task instruction type (egocentric versus object-based). However, it has been demonstrated that not the type of stimuli but the kind of instruction (left/right vs. mirrored/non-mirrored) determined, if an egocentric or object-based transformation is evoked (Voyer et al., 2017). In this study, we will focus on object-based mental rotation transformations.

## Behavioral performance in object-based mental rotation transformations

In their seminal study, Shepard and Metzler (1971) described that with abstract cube figures and object-based instructions, reaction times increase in a linear manner as a function of angular disparity. Mean reaction times increased from about 1 s with no rotation up to values between four and 6 s at 180° angular disparity for correctly answered non-mirrored pairs of cube figures. In their neuroimaging study, Jordan et. al. (2001) compared the brain mechanism and the performance in 3D cube figures with abstract shapes and letters. On the behavioral level, cube figures showed longer reaction times than the other two stimulus types. Similar to the first study, reaction times increased as a function of angular disparity. This is in line with a recently published study of Campbell et. al. (2018), who conducted an experiment with object-based transformations using Shepard and Metzler (1971) cube figures as well as human hand images. For all angles of rotation, the reaction times were higher in cube figures than for pictures of hands as stimuli.

One important matter in mental rotation research is the potential existence of sex differences. Regarding the behavioral aspects, there is a continuous discussion about whether or not and how sex influences the performance in chronometric mental rotation tests, where the test is applied on a computer and reaction time and accuracy are measured (Jansen-Osmann & Heil, 2007). If differences between males and females exist, they seem to be partially explained by task complexity and stimulus dimensionality. In three-dimensional tasks, males outperform females, whereas in two-dimensional tasks, no such difference is observed (Roberts & Bell, 2003). This gives a hint that objects, which are easier to process like embodied objects, reduced a possible sex difference. Whereas Amorim et. al. (2006) reported the processing of embodied objects in general to be easier than for abstract ones, they did not investigate sex differences. This result has been confirmed by one study of Voyer and Jansen (2016), who also examined sex differences. However, the stimulus type still showed a pronounced effect for males, i.e. males performing more accurately than females, suggesting that the use of embodied stimuli does not particularly favor females to perform better. Campbell et. al. (2018) reported contradicting results while they used cube figures and pictures of hands as stimuli. Their findings did not show any differences on the behavioral level for cube figures, but did show females outperforming males in the mental rotation of pictures of human hands. Those studies demonstrate that the topic of sex differences in chronometric mental rotation tasks is still under discussion, and if they exist, the underlying mechanisms are not well understood. One possible explanation might be that females need more cognitive effort to solve object-based mental transformation tasks (Campbell et al., 2018). One possibility to measure this cognitive effort is the use of pupillometry.

## Pupillometry in chronometric mental rotation tasks

Until now, to our knowledge, only one study investigated object-based mental rotation tasks using both stimulus types (embodied and abstract) measuring cognitive effort with respect to sex differences: Campbell et. al. (2018) implemented the physiological correlate of cognitive effort in a mental rotation task design. They compared abstract (cube) with embodied (human hand) figures in chronometric mental rotation tasks with 50 males and 49 females applying pupillometry. Pupillometry describes the measurement of the rapidly changing pupil diameter during cognitive processes. Iris dilation is regulated by the locus coeruleus-norepinephrine system mainly via norepinephrine stimulating α-adrenoceptors of the iris dilator muscle, and postsynaptic α_2_-adrenoceptors of the Edinger-Westphal nucleus that projects to the ciliary ganglion (Yoshitomi et al., 1985). Since these dilation adaptations are completely different to contractions due to the pupillary light reflex (via acetylcholine), constant low light levels are critical to reliably measure norepinephrine levels (Aston-Jones & Cohen, 2005; Koss, 1986; Nieuwenhuis et al., 2005). When set in relation to baseline values, the largest of those task-evoked changes of the pupil diameter are around 0.5 mm (Beatty & Lucero-Wagoner, 2000), and can be used as a “psychophysiological index or correlate of cognitive activity” (Campbell et al., 2018, p. 20) that changes as a result of task difficulty (Kahneman & Beatty, 1966). Kahneman (1973) used the terms ‘capacity’, ‘effort’, and ‘attention’ interchangeably to describe the limited working memory resources available to participants while solving cognitive tasks. Therefore, pupil dilation was used to measure arousal resulting from ‘cognitive load’ and it could differentiate varying difficulty between tasks. The findings of Campbell et. al. (2018) confirmed that pupil dilation was modulated by angular disparity, with higher angles increasing the pupil diameter. Additionally, females showed higher cognitive load than males in mental rotation tasks of abstract figures. For the embodied figures, for which they used hand pictures, both sexes showed comparable levels of cognitive effort, indicating that due to embodiment, sex differences in this spatial task dissipated. However, in the study of Campbell et. al. (2018), the embodied stimuli (human hands) and the cube figures differed completely in number and kind of visual features, because they did not have any shape or color features in common. A comparison might therefore be more difficult.

## Main goal of this study

To exclude the effect of different stimulus features, we used the stimuli from Amorim et. al. (2006). They created two kinds of human body figures as altered versions of the abstract cube figures. These figures differ in their similarity to the original cube figures and are labeled as body postures and human figures (Jansen et al., 2012). Using pupillometry, we also want to examine whether any differences between the stimulus types are influenced by sex, regarding cognitive load. The following hypotheses were investigated.

Sex differences in reaction time and accuracy have to be investigated. In line with Jansen-Osmann and Heil (2007), no sex differences in the behavioral data could be expected, however, with respect to the study of Voyer and Jansen (2016), males might outperform females (Hypothesis 1).

We predicted behavioral task performance to be better with embodied figures, due to the familiarity and sensorimotor functions associated with them compared to cube figures for both sexes (Hypothesis 2; Amorim et al., 2006).

According to Campbell et. al. (2018), we predicted stimulus type and angular disparity to influence the pupil dilation, with cube figures and higher angular disparity both having the highest impact. No differences between the two embodied figures were expected. We also expected reaction time to influence the changes of the pupil diameter due to the connection between task difficulty and longer response times (Hypothesis 3).

In line with the findings of Campbell et. al. (2018), we expected sex differences in pupillometric data for the abstract (cube) figures, but not for the embodied figures, with females showing higher levels in cognitive load (Hypothesis 4).

## Methods

### Participants

In total, 109 students (60 females) participated in the study and received study credits. No monetary compensation was involved in this study. In the cases of 29 participants, the Software Development Kit (SDK) had disrupted the connection to the eye tracker and thus terminated the experiment, which led to an exclusion from analysis due to software failure. This issue did not pose a threat to the data quality of all the fully completed experiments. As a result, 80 students (44 females, mean age (SD) = 20.6 (2.1) years; 36 males, 21.9 (2.8) years) form the sample for statistical analysis. To take part in the study, participants needed unrestricted eyesight at close range or corrected eyesight with contact lenses. All participants were free from eye injuries and reported no relevant physical or mental limitations. Informed consent was obtained from all individual participants included in the study. The experiment was conducted according to the ethical declaration of Helsinki. Ethical approval for this study was not required in accordance with the conditions outlined by the German Research Society (DFG), where research that carries no additional risk beyond daily activities does not require Research Ethics Board Approval. We communicated all considerations necessary to assess the question of ethical legitimacy of the study.

### Setup

Stimulus presentation and response handling were controlled with Presentation® software (Version 20.1 Build 12.04.17, Neurobehavioral Systems, Inc., Berkeley, CA, www.neurobs.com) on a Dell Latitude E5540 Laptop, 14″, 1366 × 768 px, 60 Hz. Below the bottom screen border, a RED250mobile (SensoMotoric Instruments GmbH, 2017) eye tracker, 250 Hz, was applied. Using iViewRED software (Version 4.4.26.0, SensoMotoric Instruments GmbH, 2017), all screen properties and the position of the tracker relative to the screen were integrated. With the iViewX_SDK (Version 4.4.10.0, SensoMotoric Instruments GmbH, 2017), the Presentation^®^ script conducted a 13-point calibration at the start of each run. Calibration accuracy in form of vertical and horizontal dispersion was reported to be lower than 0.3° for all participants. The iViewRED software depicts the distance of the eyes to the screen. All participants were seated close to the table. Then, the laptop was positioned with the screen being at 60 cm from participants’ eyes. No chinrest was used in this experiment. All participants were instructed to remain relaxed and to move as little as possible throughout the experiment. As a consequence, their position in the headbox of the eye tracker was always given.

The eye tracker was placed on a table, which neither the participant nor the investigator touched during the experiment to control for vibrations on the tracker. To maintain an appropriate distance to the participant and to manage the software, the investigator used a wireless keyboard and mouse on a separate table. The participant used a wired mouse placed on a lower table beneath the tracker table to control for input lag and vibrations. The laboratory was silent and constantly dimly lit to control for pupil dilation due to light conditions. The only light sources were a ceiling lamp outside the peripheral visual field of the participant and the laptop screen (luminance at 169 cd/m^2^ for body postures, and 177 cd/m^2^ for both cube and human figures; measured with a spot meter, Chroma Meter CS-100, Minolta Co., Ltd., Japan), resulting in a constant illuminance of 55 lx (measured with a lux meter, testo 540, Testo AG, Germany).

### Stimuli

**Fig. 1:**
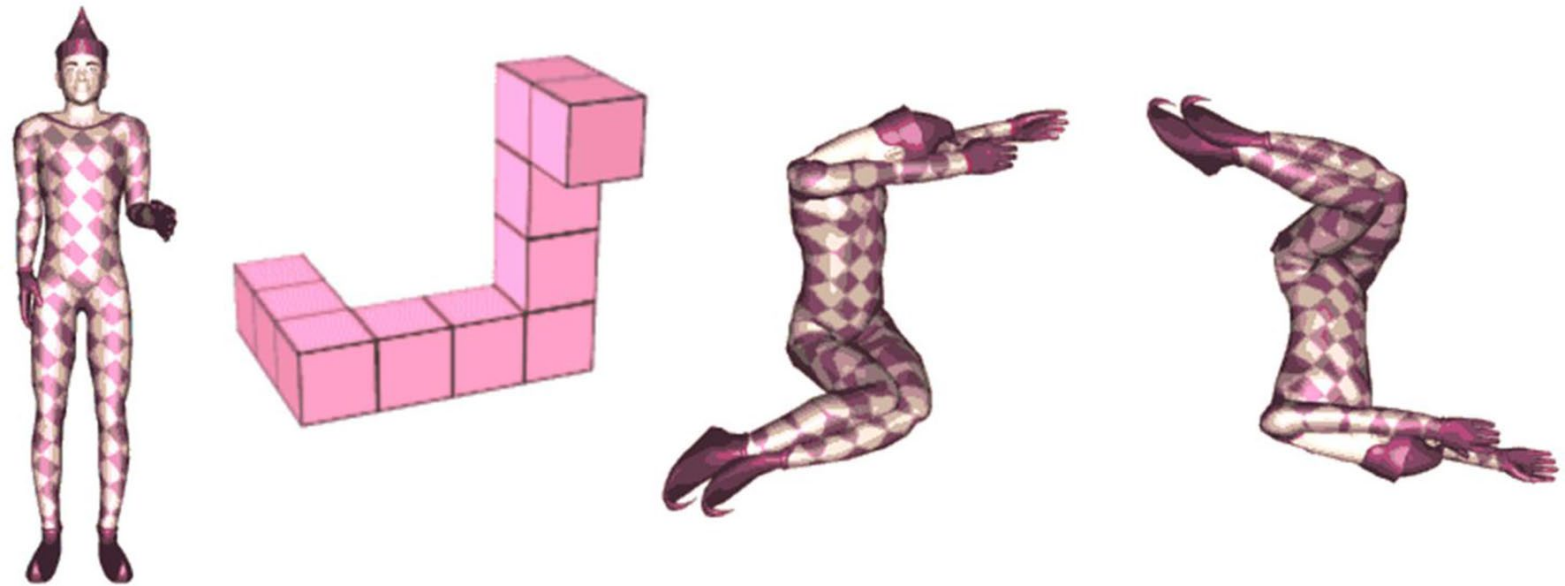
Examples of mental rotation stimuli used in the experiment (developed by Amorim et al., 2006; reprinted and adapted with permission by Michel-Ange Amorim). From left to right: human figure (non-mirrored). Cube figure (non-mirrored). Body posture (non-mirrored). Body posture (mirrored and rotated by 180°)

Stimuli consisted of three different types developed by Amorim et. al. (2006), which are Shepard-Metzler style 3D cube figures, human figures, and body postures (see Fig. 1). Human figures are human bodies in standing positions holding their arms in different positions, matching familiar postures (e.g. shaking hands). Body postures are human bodies, whose atypical postures aligned with the cube figure configurations. All stimulus types had three variations each, were partially colored in pink, and were displayed in front of a white background. The participants looked at two three-dimensional figures (pairwise) and had to decide whether the figures were the same or different (mirrored). Each figure type was presented in a separate block (three blocks in total) with 42 trials each (total of 126 randomized trials) with one half of the trials being identical pairs and the other half mirror-reversed pairs. On the left side of the screen, the model was always presented non-mirrored with 0° rotation. On the right side of the screen a rotated and mirrored/non-mirrored stimulus was presented. The stimuli were presented in seven different angular disparities of 0°, 30°, 60°, 90°, 120°, 150° and 180° in *y*-axis (screen plane). Each figure had a dimension of 400 × 400 px and was vertically centered and horizontally positioned 300 px to the left or right of the center of the screen until a response was given. A practice block of 36 trials with feedback preceded the main experiment. Between stimuli pairs in the practice session, participants received feedback for 1000 ms (+ right, − wrong) shown at the center of the screen, and in experimental sessions, a fixation cross (“*”) was shown there for 1000 ms. During the main experiment, self-controlled pauses were provided after every 14 trials.

### Pupil diameter

Before the practice block, the participants saw each stimulus pair of all nine figures in randomized order and only for the 0° non-mirrored condition. Participants were instructed that they were about to see some pictures, which they only had to look at. Originally, this first block was supposed to serve as baseline measurements, as Campbell et. al. (2018) used them, for instance. However, we followed the recommendations by Mathôt and colleagues (Mathôt, 2013; Mathôt et al., 2015, 2018), and used a trial-dependent baseline correction. The response in pupil dilation typically occurs within the first few hundred milliseconds after stimulus onset, peaks around 1–2 s after stimulus onset and continues asymptotically until it returns back to baseline values (Andreassi, 2000; Beatty & Lucero-Wagoner, 2000; Loewenstein & Loewenfeld, 1962; Nieuwenhuis et al., 2011). More specifically, task-evoked pupil responses do not emerge earlier than ca. 220 ms after a manipulation that caused them (e.g., Mathôt et al., 2015). This opens up the possibility to take a period at the start of each trial for baseline correction. For this mental rotation experiment, we followed the approach of Mathôt et. al. (2018) and used the median pupil size during the first eleven samples (corresponding to 40 ms), which was then used for subtractive baseline correction for each trial. Subtractive baseline correction is favorable, because it is more robust and increases statistical power more than divisive correction (see, Mathôt et al., 2018, for an in-depth comparison). Overall, the baseline correction served to decrease the impact of random pupil-size fluctuations from one trial to the next, whereas between subject differences were taken into account statistically by using by-participant random intercepts in the linear mixed models (see Baayen et al., 2008; Mathôt et al., 2018).

Statistical inspection of the baseline data showed no significant differences between the angles (*p* = 0.073), but significant differences between the stimulus types (*p* = 0.007). Pairwise comparisons indicated that the pupil diameters for body postures were larger than for cube and human figures (which did not differ significantly from each other), illustrating the luminance differences between those stimuli.

### Procedure

The experiment was a single session and lasted between 35–50 min, depending on participants’ speed to complete all items. Upon arrival, the participants read and signed the informed consent. After that, they filled out a questionnaire including demographic information, sports activity, physical and mental illnesses, and eye-sight specifications. Then, they were positioned, as was the laptop respectively. After a brief explanation of the test protocol, the calibration and first block (presenting nine non-mirrored and non-rotated stimulus pairs for 6 s each) were run. Then the practice session with feedback followed, which was introduced by a digitally presented instruction. Participants used the right hand for mouse handling and received written instructions to press the left mouse button, if the stimuli could be rotated into congruence (non-mirrored, “same”), and the right mouse button, if the two stimuli were mirrored (“different”), and to answer as quickly and precisely as possible. Here, verbal feedback was only given, when participants did not understand the task, hence overly making mistakes or taking very long to respond (> 15 s). After completing all practice trials, the calibration and main session were run. During the practice and main sessions, participants were asked to remain with their gaze on the screen (being allowed to blink naturally). During the short self-controlled breaks, they could avert or close their eyes for a few seconds. These instructions were necessary, because the SDK would produce an error and shut down the experiment upon longer gaze losses. Following the main experiment, the participants were debriefed. All verbal instructions were standardized using a researchers’ guideline script. Three investigators conducted the data collection.

### Study design

To analyze cognitive performance, the dependent variables are reaction time (RT), accuracy (ACC) as well as the difference between the maximum pupil diameter and the respective baseline pupil diameter for each trial (PD) as a measure of cognitive load. To test our hypotheses, the independent variables are stimulus type (STI; cube figures [CF], human figures [HF], and body postures [BP]), SEX, angular disparity (DEG), and their respective interactions. DEG describes the angular disparity between the two figures shown on the screen. Since the left image is always presented with 0° rotation, the angular disparity depicts the rotation in degrees of the right image. DEG was included as a fixed effect as it is the main moderator of difficulty in mental rotation tasks (Jost & Jansen, 2020). In the analysis of PD, we included RT as a fixed effect to analyze the influence of reaction time on cognitive load.

### Data processing

For the behavioral data, outliers were determined by a deviance of more than three standard deviations from the mean reaction time of all stimulus pairs with the same rotation angle and were excluded from all analyses. Because angular disparity is not defined for mirrored responses in cube figures (Jolicœur et al., 1985; Shepard & Metzler, 1971), only non-mirrored stimulus pairs were analyzed and reaction time was additionally only analyzed for correct responses. Using the SMI software BeGaze 3.7, build 58 (SensoMotoric Instruments GmbH, 2017), a velocity dependent algorithm (peak velocity threshold = 40°/s, min. fixation duration = 50 ms, peak velocity between 20–80% of saccade length) was used for blink detection. Here, blinks are saccade-like events during which fast pupil diameter changes occur (which reflects a rapid shrinking of the pupil due to closing of the eyelid). In R (R Core Team, 2018), the exported raw data (with marked blinks) were further processed to obtain valid pupil diameter data. The data for each eye were treated separately. As recommended by Mathôt et. al. (2018), in addition to filtering based on detected blinks, we also filtered based on pupil size. A band pass filter was used to reject pupil size samples outside a predefined range between 1.5 and 9 mm (Kret & Sjak-Shie, 2019; Kret et al., 2014). Based on Mathôt’s (2013) approach, we reconstructed the pupil sizes for blinks (each window extended by 10 ms before and after) as well as gaps, using cubic spline interpolation. Beforehand, we filtered trials that had no pupil size data during the first or last 20 ms of the trial, so that sufficient data would be available for the baseline. The last 20 ms were chosen to keep the data symmetrical, and because the highest peaks in cognitive load would likely occur shortly before the task response, taking into account the delay in pupil dilation (see above, Mathôt et al., 2015). After that, the median pupil size during the first 40 ms was calculated as the baseline value for each trial (see, Mathôt, 2013). A 10-point moving average filter was run to smooth the data for noise, the maximum pupil size was determined for each trial, and the difference to the baseline was calculated. As Beatty and Lucero-Wagoner (2000) stated, most of the task-induced pupil size changes are below 0.5 mm. To also account for rarer cases, we excluded differences larger than 0.6 mm. After that, the pupil size change was averaged between the two eyes. In case of available data for only one eye, this value was taken, due to the diameters of both eyes being highly correlated, especially locally (Jackson & Sirois, 2009). Overall, out of 10,080 trials, 233 had missing data from both eyes and were excluded from analysis. In line with Campbell et. al. (2018), we analyzed the maximum pupil diameter changes according to common behavioral data analysis, i.e. excluding mirrored items and wrongly answered ones.

### Statistical analysis

Statistical analysis was performed using *lme4* package (Bates et al., 2015a, 2015b) in R (R Core Team, 2018). Reaction time and pupil diameter were analyzed using linear mixed models and accuracy was analyzed using generalized linear mixed models with a binomial distribution. Model parameters were estimated by maximum likelihood estimation. *p*-values were obtained by using likelihood ratio tests to test for improvement of model fit by the fixed effect of interest and compared to a significance level of 0.05. Confidence intervals were calculated using parametric bootstrapping with 1000 simulations. Visual inspection of residual plots did not reveal deviations from homoscedasticity or normality in any model.

Hypothesis-driven model building was based on the research of Barr et. al. (2013), and Bates et. al. (2015a, 2015b), starting with a model with random intercepts and slopes for every appropriate fixed effect and reducing the model complexity by dropping non-significant variance components. Non-significant fixed effects were further removed from the model, such that non-significant effects were tested for an improvement of model fit by inclusion in the resulting model while significant effects were tested for worsening of model fit by exclusion of the effect. Main effects for significant interactions were tested separately by splitting the interaction (also see, Jost & Jansen, 2020). The resulting models for each parameter are described in the results section. All data were visualized using *ggplot2* package (Wickham, 2016) in R (R Core Team, 2018).

## Results

### Reaction time

**Fig. 2:**
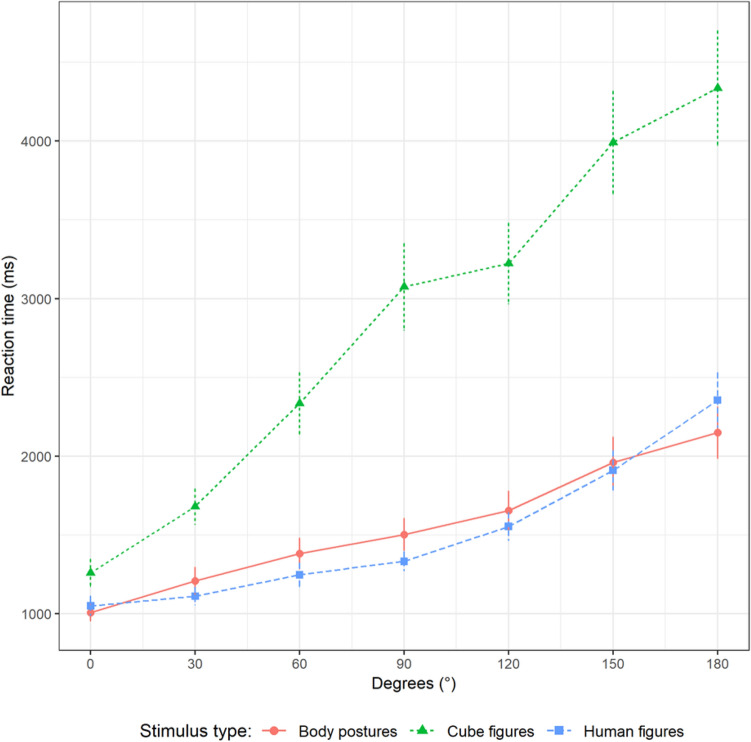
Reaction time plotted against angular disparity and stimulus type

As shown in Fig. 2, the reaction times for cube figures are constantly higher than for body postures and human figures. The graphs of the two embodied stimulus types are more closely related. All graphs show a positive slope with increasing angular disparity.

Model construction resulted in a model with random intercepts and slopes for STI and DEG by participant. STI*SEX*DEG and all respective interactions and main effects were analyzed as fixed effects. Significant differences were found for STI*DEG (see Table 1). Reaction time increased significantly by DEG and STI (main effects). The interaction DEG*STI showed a significant increase in reaction time with increasing DEG for all stimulus types, with CF having the highest increase, followed by HF and BP. Pairwise comparisons for the interactions showed significant differences between all of them. Pairwise comparisons for the main effects showed significant differences between CF and BP, and CF and HF, but not for HF and BP, with CF always having higher values.

**Table 1:** Statistical analysis of reaction time (in seconds)

Variable	Estimate	SE	Test statistic	*p* value	95% CI
Intercept	1.00	0.05			0.89, 1.10
DEG*STI			*χ*^2^(2) = 522.33	< 0.001	
DEG*STI(BP)	0.59	0.05			0.49, 0.68
DEG*STI(HF-BP)	0.09	0.04	*χ*^2^(1) = 7.29	< 0.001	0.01, 0.18
DEG*STI(CF-BP)	1.02	0.05	*χ*^2^(1) = 367.87	< 0.001	0.93, 1.11
DEG*STI(CF-HF)	0.93	0.05	*χ*^2^(1) = 301.95	< 0.001	0.83, 1.03
DEG(0°)*STI(HF-BP)	− 0.12	0.06			− 0.23, − 0.01
DEG(0°)*STI(CF-BP)	0.19	0.08			0.03, 0.35
Main Effects					
DEG (100°)	0.91	0.04	*χ*^2^(1) = 151.84	< 0.001	0.83, 1.00
STI			*χ*^2^(2) = 105.33	< 0.001	
STI(HF-BP)	− 0.03	0.04	*χ*^2^(1) = 0.89	0.345	− 0.11, 0.04
STI(CF-BP)	1.03	0.07	*χ*^2^(1) = 103.22	< 0.001	0.90, 1.17
STI(CF-HF)	1.07	0.08	*χ*^2^(1) = 100.42	< 0.001	0.03, 1.21
Non-significant Effects					
SEX (male–female)	0.02	0.06	*χ*^2^(1) = 0.09	0.762	− 0.10, 0.14
SEX*DEG	0.09	0.07	*χ*^2^(1) = 1.47	0.225	− 0.06, 0.22
SEX*STI			*χ*^2^(2) = 2.05	0.360	
SEX*STI*DEG			*χ*^2^(2) = 4.11	0.128	

Intercept in this model represents the estimate at 0° for body postures (BP). Effects of angular disparity (DEG) represent changes of 100°. Test statistic and *p*-value for stimulus type (STI) and DEG*STI represent the pairwise comparisons (*HF* human figures; *CF* cube figures)

Regarding reaction time and our first hypothesis, sex differences emerged neither for the main effect (SEX) nor for the interactions. The results support our second hypothesis that mental rotation task performance would be better with embodied figures, which is shown both by the overall difference between the reaction times of the two embodied figure groups and the abstract figure one (STI; highest for CF) as well as the increase of reaction time for each degree of angular disparity (DEG*STI; highest for CF).

### Accuracy

**Fig. 3:**
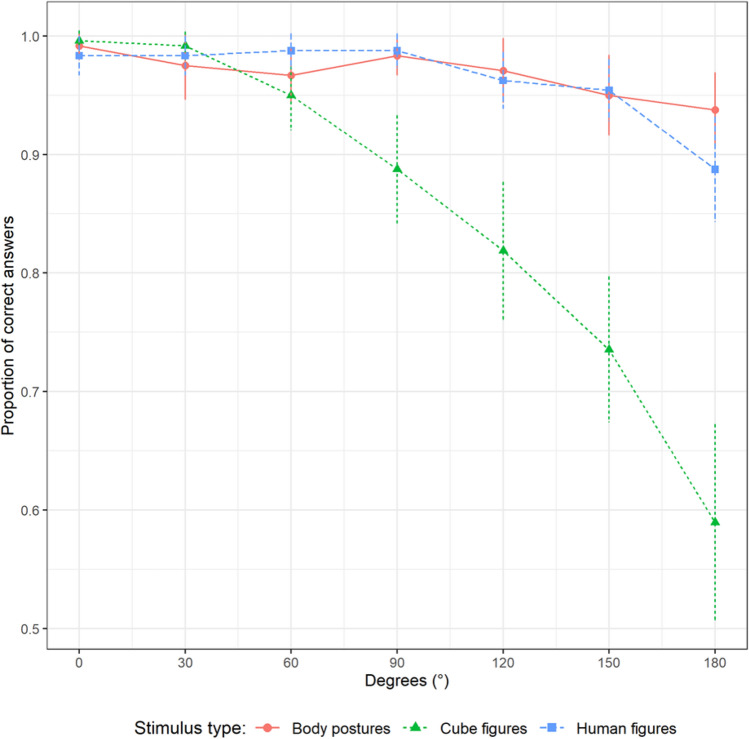
Accuracy plotted against angular disparity and stimulus type

In Fig. 3, the proportion of correct answers shows a steeper decline for cube figures than for body postures and human figures. The graphs of the two embodied stimulus types are more closely related. All graphs start similarly at zero degrees and show a negative slope with increasing angular disparity.

Model construction for ACC resulted in a model with random intercepts and slopes for STI and DEG by participant. STI*SEX*DEG and all respective interactions and main effects were analyzed as fixed effects. Significant differences were found for STI*DEG (see Table 2). Accuracy decreased significantly by DEG and STI (main effects). The interaction DEG*STI showed a significant decrease in accuracy with increasing DEG for all stimuli, with CF having the highest decrease, followed by HF and BP. Pairwise comparisons for the interactions showed significant differences between CF and BP, and CF and HF (with CF having larger decreases), but not for HF and BP. Pairwise comparisons for the main effects showed significant differences between all of them (with CF having lower values).

**Table 2:** Statistical analysis of (logarithmic odds of) accuracy

Variable	Estimate	SE	Test statistic	*p* value	95% CI
Intercept	5.47	0.58			4.54, 7.07
DEG*STI			*χ*^2^(2) = 14.36	< 0.001	
DEG*STI(BP)	− 1.04	0.35			− 1.85, − 0.36
DEG*STI(HF-BP)	− 0.53	0.45	*χ*^2^(1) = 2.87	0.090	− 1.46, 0.35
DEG*STI(CF-BP)	− 1.36	0.40	*χ*^2^(1) = 9.65	0.002	− 2.23, − 0.55
DEG*STI(CF-HF)	− 1.03	0.41	*χ*^2^(1) = 6.33	0.012	− 1.84, − 0.24
DEG(0°)*STI(HF-BP)	− 0.15	0.71			− 1.67, 1.18
DEG(0°)*STI(CF-BP)	− 0.67	0.64			− 2.23, 0.42
Main Effects					
DEG (100°)	− 1.95	0.18	*χ*^2^(1) = 94.15	< 0.001	− 2.33, − 1.61
STI			*χ*^2^(2) = 66.44	< 0.001	
STI(HF-BP)	− 0.93	0.41	*χ*^2^(1) = 5.24	0.022	− 2.15, − 0.21
STI(CF-BP)	− 2.58	0.39	*χ*^2^(1) = 64.54	< 0.001	− 3.72, − 1.96
STI(CF-HF)	− 1.69	0.25	*χ*^2^(1) = 46.50	< 0.001	− 2.31, − 1.23
Non-significant Effects					
SEX (male–female)	0.24	0.21	*χ*^2^(1) = 1.29	0.256	− 0.18, 0.59
SEX*DEG	− 0.05	0.31	*χ*^2^(1) = 0.03	0.863	− 0.68, 0.56
SEX*STI			*χ*^2^(2) = 2.22	0.330	
SEX*STI*DEG			*χ*^2^(2) = 1.21	0.545	

Intercept in this model represents the estimate for the logarithmic odds at 0° for body postures (BP). Effects of angular disparity (DEG) represent changes of 100°. Test statistic and *p*-value for stimulus type (STI) and DEG*STI represent the pairwise comparisons (*HF* human figures; *CF* cube figures)

With regard to accuracy and our first hypothesis, sex differences emerged neither for the main effect (SEX) nor for the interactions. The results also support our second hypothesis that task performance would be better with embodied figures, which is shown both by the overall difference between the accuracy of the two embodied figure groups and the abstract figure one (STI; lowest for CF) as well as the decrease of accuracy for each degree of angular disparity (DEG*STI; largest for CF). The results are similar to those for reactions times, except for the pairwise comparison of the main effect (STI(HF-BP); significant differences in accuracy, but not in reaction time) and the slope by DEG (DEG*STI(HF-BP); significant differences in reaction time, but not in accuracy) between the two embodied figure groups.

### Pupil diameter

**Fig. 4:**
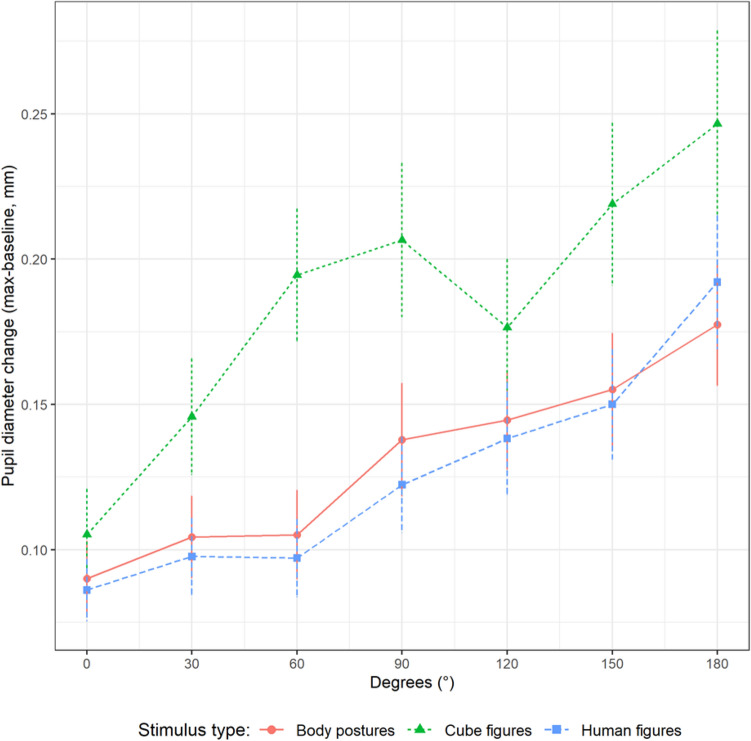
Changes of pupil size (max-baseline) plotted against angular disparity and stimulus type

The task-evoked pupil responses are depicted in Fig. 4 and show an inclination of all graphs with increasing angular disparity. Here, cube figures show constantly higher values than the two embodied figure types. The graphs of body postures and human figures lie closer together.

The model building resulted in a model with random intercepts and random slopes for DEG and RT by participant. STI*SEX*DEG, RT, and all respective interactions and main effects were analyzed as fixed effects. The pupil diameter increased significantly by STI*DEG, and RT (see Table 3). Pupil size increased significantly by DEG and STI (main effects). The interaction STI*DEG showed a significant increase in pupil size for each DEG for the different stimuli, with HF having the highest increase, followed by BP and CF. Pairwise comparisons for the interactions showed significant differences between CF and HF, but not for CF and BP, and HF and BP. Pairwise comparisons for the main effects showed significant differences between CF and BP, and CF and HF, but not for HF and BP. With regard to the inclusion of RT in the model, we inspected the data for possible collinearity problems. All variance inflation factors were smaller than three (maximum of 1.86), i.e. collinearity was not an issue (see, Zuur et al., 2010).

**Table 3:** Statistical analysis of pupil diameter (in 10^–1^ mm)

Variable	Estimate	SE	Test statistic	*p* value	95% CI
Intercept	1.41	0.06			1.27, 1.53
DEG*STI			*χ*^2^(2) = 8.72	0.013	
DEG*STI(BP)	0.28	0.05			0.19, 0.37
DEG*STI(HF-BP)	0.06	0.06	*χ*^2^(1) = 0.71	0.399	− 0.06, 0.17
DEG*STI(CF-BP)	− 0.13	0.06	*χ*^2^(1) = 1.33	0.250	− 0.25, 0.00
DEG*STI(CF-HF)	− 0.16	0.07	*χ*^2^(1) = 5.98	0.015	− 0.30, − 0.03
Main Effects					
RT (sec)	0.37	0.03	*χ*^2^(1) = 97.59	< 0.001	0.31, 0.43
DEG (100°)	0.28	0.04	*χ*^2^(1) = 45.74	< 0.001	0.21, 0.35
STI			*χ*^2^(2) = 40.17	< 0.001	
STI(HF-BP)	− 0.03	0.03	*χ*^2^(1) = 0.64	0.423	− 0.09, 0.04
STI(CF-BP)	0.22	0.04	*χ*^2^(1) = 35.09	< 0.001	0.14, 0.30
STI(CF-HF)	0.27	0.04	*χ*^2^(1) = 39.18	< 0.001	0.18, 0.36
Non-significant Effects					
SEX (male–female)	− 0.09	0.08	*χ*^2^(1) = 0.10	0.752	− 0.25, 0.07
SEX*DEG	− 0.09	0.07	*χ*^2^(1) = 2.82	0.093	− 0.23, 0.05
SEX*STI			*χ*^2^(2) = 5.47	0.065	
SEX*STI*DEG			*χ*^2^(2) = 0.15	0.928	

Intercept in this model represents the estimate for body postures (BP), average reaction time (RT), and average angular disparity (DEG). Effects of DEG represent changes of 100°. Test statistic and *p*-value for stimulus type (STI) represent the pairwise comparisons (*HF* human figures; *CF* cube figures)

In the results for changes of pupil size, there was evidence for our third hypothesis that the pupil dilation would be influenced by the stimulus type and angular disparity. This is shown both by the increase of the pupil size change with increasing angular disparity (DEG*STI) as well as the overall difference between the pupil size change of the two embodied figure groups and the abstract figure one (STI). Here, the abstract figures have the lowest slope for DEG*STI, which could partially result from their constantly higher values in pupil size change (STI). There was no evidence for our fourth hypothesis, as no sex differences emerged neither for the main effect (SEX) nor for the interactions.

### Exploratory results

We exploratively analyzed the time course of pupil dilation throughout the trials. Next to gaining insight into time-dependent pupil size changes of interest for future studies, this can also help to check the validity of the data, both as a check for the selected pupil size change measures (baseline and maximum) as well as possible disturbances by e.g. carry-over effects.

**Fig. 5:**
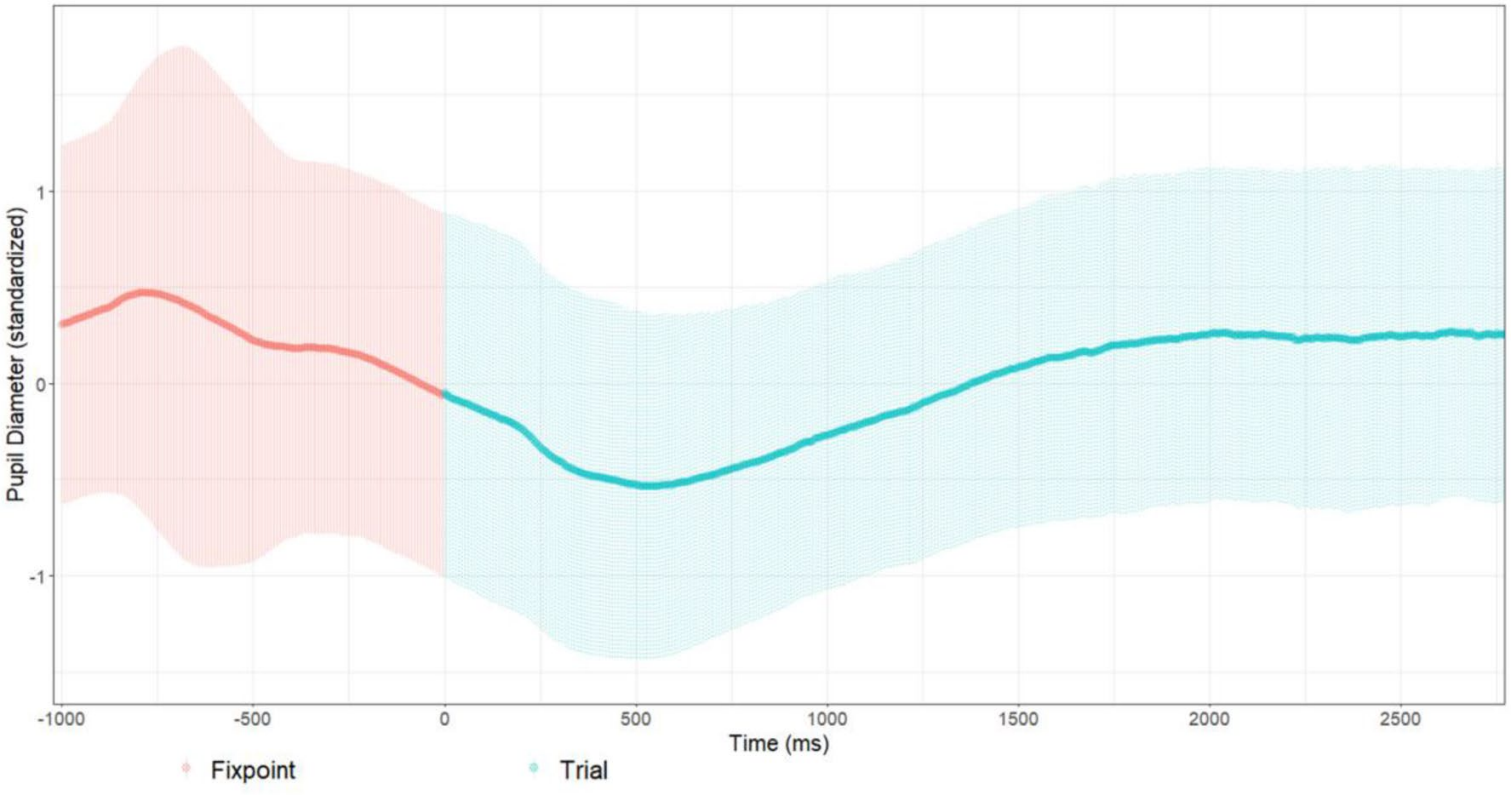
Time course of the standardized pupil diameter means of all participants’ trials, depicting only the first 3 s; data of shorter trials only go until their respective trial end. Zero represents the onset of the stimulus preceded by the fixation point (− 1000–0 ms). The shaded areas represent plus/minus one standard deviation

**Fig. 6:**
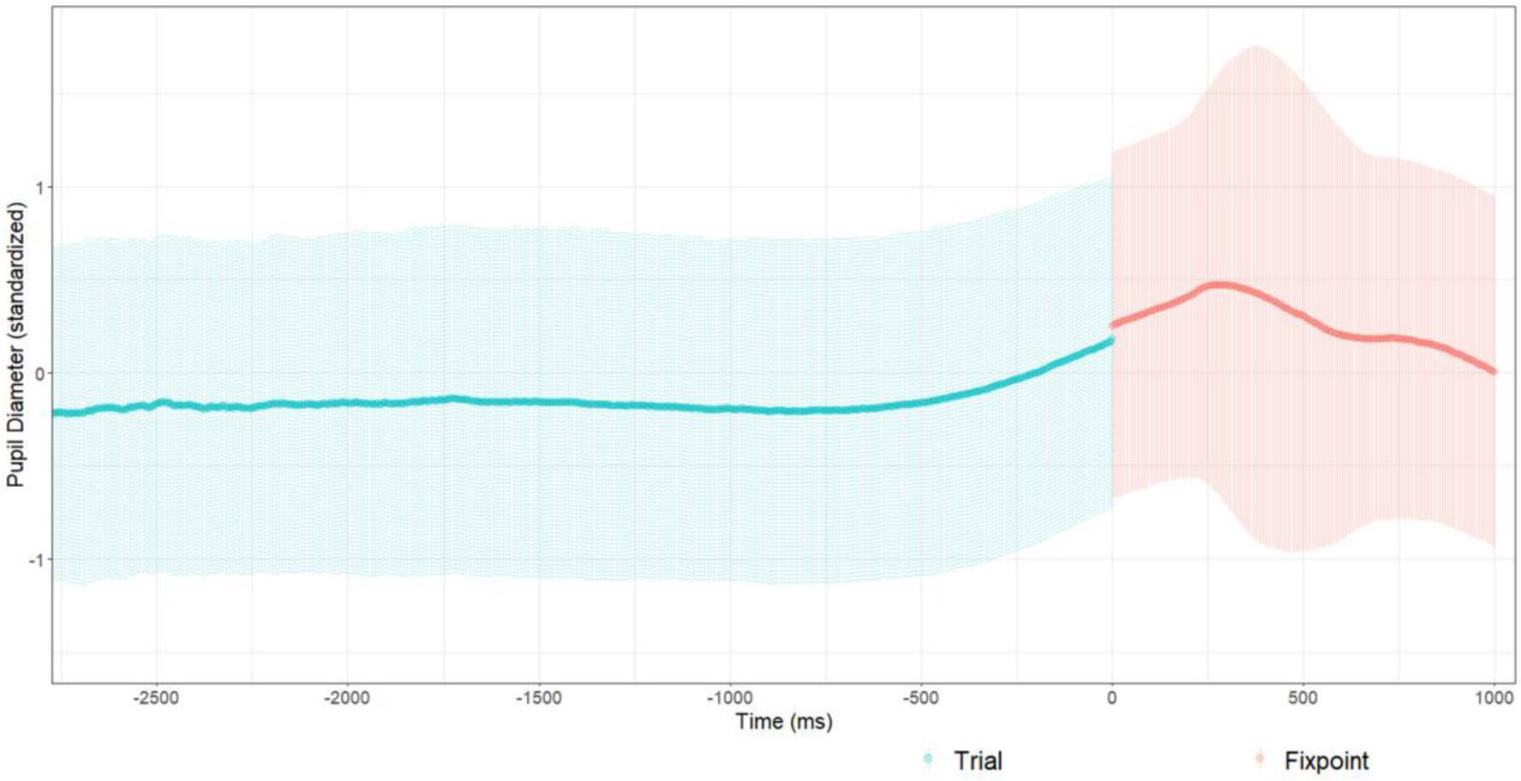
Time course of the standardized pupil diameter means of all participants’ trials, depicting only the last 3 s; data are aligned for trial ends at 0 ms; data of shorter trials only begin at their respective trial start. Zero represents the end of the stimuli and is followed by the fixation point (0–1000 ms). The shaded areas represent plus/minus one standard deviation

The time course of pupil dilation (see Fig. 5) illustrated that the pupil size decreased after trial start for around 500 ms and began to increase thereafter. Alignment of the trial ends illustrated that the pupil diameter increased during the time around task response, leading to higher values during the fixation point (see Fig. 6).

## Discussion

### Validity of pupil size measurements

Before addressing the hypotheses, we would like to elaborate on the temporal analysis of the pupil size means. Visual inspection of the time course of pupil dilation indicated possible carry-over effects. Over all trials, pupil size decreased after trial start for 500 ms and began to increase thereafter. Therefore, the one second fixation point duration was seemingly not enough for the pupil diameter to fully return to its baseline value. Inspecting the end of trials indicated that the pupils were still dilating before, while, and after task response (mouse-click) was given, leading to higher values during the fixation point. This is a possible concern to the validity of the measurements as the exact extent and duration of the observed effects are unknown. One possible explanation is that shorter trials were systematically affected more, as the recording was cut off earlier and the trailing pupil dilation would lead into the following fixation point and trial. On longer trials, there was more time for the pupil size to plateau, which could be a reason for our results indicating that higher values emerged for higher angular disparity. This explanation however is unlikely, as this should have been compensated by the included effect of reaction time on pupil dilation. Nevertheless, assuming that carry-over effects only depend on the difficulty of the previous trial (and trials are in random order) and are somewhat random in magnitude and duration, they at least introduce additional variance to the measurements, which in turn reduces the power of the design.

Despite these issues, we did not choose to alter the measurements. Regarding the baseline measurement, we cannot isolate a baseline due to the possible overlap in time of the observed decrease in pupil size and the expected increase due to the cognitive effort. However, we also conducted the pupil diameter analysis using baseline values for each stimulus, which were recorded at the beginning of the experiment. This form of analysis has its own problems, i.e. mainly having no control of the random trial by trial pupil fluctuations. Interestingly, this and our described analysis results did not differ in the significance of any effect in question. This could indicate that the starting values were overall only shifted, i.e. baseline measurements at the start of each trial were always 0.5 units larger than the true baseline. Regarding the measurement of maximal pupil dilation, one could include the following fixation point in the analysis of each trial. This however would have other effects influencing the data, e.g. new visual input and pupillary light reflex. In addition, altering the analysis would not change the problem of the carry-over effects. In consequence, regarding the hypotheses for the pupillometric measurements, the results must be considered with caution, independent of a possible change of measurements. Nevertheless, these issues are important for both past and future studies of pupil dilation during mental rotation and also other cognitive tasks where similar problems might arise. In experiments where the trial was cut off directly after task response, the interpretation of the results has to be done cautiously, as carry-over effects might have a similar impact there (e.g. in, Campbell et al., 2018). In a recent study using another approach, Bochynska et. al. (2021) showed the stimulus for 4 s, independent of task response. However, since trials with response times longer than 4 s (141 of 1064 trials) were excluded from the analysis, tasks of higher angular disparity might only have been partially included, and the ones included could also have faced the problems of delayed pupil dilation.

Based on the observed time course of pupil dilation, future studies should (1) keep showing the task and measuring pupil dilation even after the response for at least 500 ms, and (2) increase the break between trials to at least 2 s.

### General discussion

The results show no sex differences in the behavioral performance. The main effects and interactions for both accuracy and reaction time do not show any influence of sex on the models. This is in line with other chronometric mental rotation studies. For instance, Voyer et. al. (2006) also report no sex differences in mental rotation performance of 3D cube figures. Jansen-Osman and Heil (2007) investigated sex differences in mental rotation tasks with five different stimulus types and also reported sex differences only in one (polygons) of these (3D cube figures, letters, stimuli from primary mental abilities, and animal pictures). However, the results are in contrast to the study of Voyer and Jansen (2016)—using stimuli that were partially the same as in this study—who pointed out that although a performance improvement for both sexes was apparent, males might benefit more from the advantage through embodiment. One reason for this discrepancy may be the variation in the use of the human stimuli between the two studies. Voyer and Jansen (2016) presented head cubes (cubes with the addition of a head) while we investigated human postures.

In accordance with Amorim et. al. (2006), our results confirm our second hypothesis that task performance would be better for both embodied figures compared to cube figures for both sexes. Our experiment shows a significant embodiment effect. Both embodied figures in this object-based transformation task were processed more easily on a behavioral level (shorter reaction time and higher accuracy), matching our hypothesis. This is in congruence with other studies (e.g. Amorim et al., 2006; Campbell et al., 2018; Voyer & Jansen, 2016).

In line with the paradigm for chronometric mental rotation tasks, changes in angular disparity significantly influenced all dependent variables for all models. Higher angular disparity between the two pictures resulted in higher reaction times and lower accuracy. These effects are larger for the abstract than for the embodied figures. In particular, the interaction of cube figures and angular disparity showed a higher negative impact on performance than for body postures and human figures.

In terms of cognitive load (hypothesis 3), cube figures show the highest values in pupil diameter, followed by body postures and human figures. Here, both embodied figure types differ significantly from the cube figures. Therefore, the highest cognitive load manifests in cube figures, indicating that these tasks are more difficult to solve. This finding is congruent with our results for behavioral performance.

An additional possible explanation for the pupil diameter to be lower for both embodied figures than for cube figures might be a congruency effect. In a pupillometry experiment regarding the Stroop task, Hershman and Henik (2019) report lower pupil diameter values for neutral (colored letters with no meaning) than for color-congruent (word and word color align) tasks, indicating higher cognitive load in the latter due to a task conflict between reading the word and naming the color of the word. This is based on the effect that stimuli evoke tasks, which are strongly associated with them (Rogers & Monsell, 1995; Waszak et al., 2003). That means the neutral colored word has more task congruency, because it only elicits naming the color, whereas the colored word elicits reading of the word, creating conflict with the response. These findings concur with the description of motoric embodiment (i.e. imagination and execution of actions addressing the same motor representations) of Amorim et. al. (2006), and might also apply to this study. Task response was given in interaction with a desktop mouse. With the hand being an important and salient feature of human figures and body postures, a congruency effect with the hand response is possible. That is, higher congruency could also lead to lower cognitive load and might thus be an additional factor considering motoric embodiment effects in this experiment.

Overall, the first part of our hypothesis 3 was confirmed by the modeling results, with the main effects of cube figures and higher angular disparity increasing the pupil dilation the most. Also as predicted, the two embodied figure types did not differ significantly. Interestingly, the interaction of stimulus type and angular disparity showed the highest increases for human figures. With the implementation of reaction time in the model, the results illustrate the additional effect of this interaction on top of the effect of reaction time. Here, the pupil sizes for cube figures are higher due to the higher difficulty to solve the task. Thus, the range to increase was smaller than for the embodied figures, resulting in a less steep slope by angular disparity, which might indicate a ceiling effect in this regard. However, no significant differences emerged between body postures, and cube and human figures, with the values of body postures lying between those two. Consequently, they cannot be placed properly in this regard, which should be further investigated in future experiments. Additionally, changes in pupil dilation did not get fully explained by variations in angular disparity. Reaction time itself still predicted a significant portion of the pupil diameter in the statistic model. That is, both reaction time and angular disparity had an impact on cognitive load, but none of them alone seemed to be sufficient to describe the connection between each other, and to account for task difficulty. As a consequence, it is possible that the relationship between difficulty and cognitive load is not linear.

Our expectations regarding the sex differences in pupil dilation were based on the findings of Campbell et. al. (2018), but our results did not confirm these predictions, as no differences emerge. As a conclusion, both sexes showed similar performance in the mental rotation tasks and exerted the same levels of cognitive effort for each stimulus type. This also means that all embodiment facilitations provoked similar changes in cognitive load and task performance for both sexes. This gives a hint that both males and females are able to solve mental rotations tasks, which have the same amount of spatial embodiment, with the same effort (Amorim et al., 2006). Spatial embodiment includes the mapping of a body-relevant coordinate system that facilitates the mental rotation process. In our case, having controlled object-based transformations as instructed, spatial embodiment seems to partially explain the differences in behavioral performance. With the results for pupil dilation matching those for behavioral data, spatial embodiment can explain the reduced pupil size for embodied figures, having a logical alignment in the geometric space.

Our findings did not show any differences between the two embodied stimulus types. This is interesting, as they differ between each other regarding their geometric form. Body postures and cube figures are s-shaped comprising three bends, with body postures imitating the shape of the abstract figures. Human figures instead comprise only one or two bends, and are more i- or t-shaped. Thus, the stimulus types differ in spatial complexity, and human figures could theoretically be assumed to facilitate visual absorption and processing. However, the difference in geometric form had no impact in this experiment. Overall, our results provide—for the first time—evidence that males and females need the same cognitive effort for the solution of this task.

## Limitations

In pupillometry studies, the pupil foreshortening error should be of concern to have an influence on the acquired data, especially when looking at areas, which are further away from the screen center (Hayes & Petrov, 2016). In our mental rotation tasks, all analyzed image pairs are shown at the same positions on the screen. Possible pupil foreshortening errors were not expected to systematically differ between conditions, and were neglectable for that reason (see, Mathôt et al., 2018).

Amorim et. al. (2006) neither analyzed sex differences nor elaborated on their choice of color. The partially pink coloring of the stimuli might facilitate female performance due to possible familiarity effects. However, a similar effect could also apply to males in respect to cube figures (Ruthsatz et al., 2017). To further analyze effects of stimulus color, the same stimuli would have to be presented in different color schemes (e.g. black–white, blue, and pink), and analyzed regarding sex differences.

In this experiment, no chinrest was used. Although the SMI software is able to compensate for variations in head-tracker distance, these estimations might have limitations. Those can result in random noise, which does not endanger the validity of the results, but could be avoided nonetheless. Thus, in pupillometry experiments, using a chinrest is recommendable.

As mentioned in the discussion, the experimental design led to possible carry-over effects, which have to be taken into account for interpretation of the results.

## Conclusion

This study replicated the effect for mental rotation tasks of embodied stimuli to be easier to process (Amorim et al., 2006) and showed for the first time that this result is confirmed with pupillometry, meaning that mental rotation of embodied figures needs less cognitive effort to solve the task. Sex differences did not appear in any of the measurements.

## Data Availability

The data that support the findings of this study are available from the corresponding author on reasonable request.

## Code availability

The Presentation^®^ (Neurobehavioral Systems, Inc., Berkeley, CA, www.neurobs.com) and R (R Core Team, 2018) code used for this study is available from the corresponding author upon reasonable request.
